# Supplementary material for: p38-MAPK-mediated translation regulation during early blastocyst development is required for primitive endoderm differentiation in mice
Source: Commun Biol. 2021 Jun 25;4:788. doi: 10.1038/s42003-021-02290-z (PMC8233355; doi:10.1038/s42003-021-02290-z)
Supplement: Supplementary file 2 — Supplementary Information [file 42003_2021_2290_MOESM2_ESM.pdf]

Supplementary Fig. 1

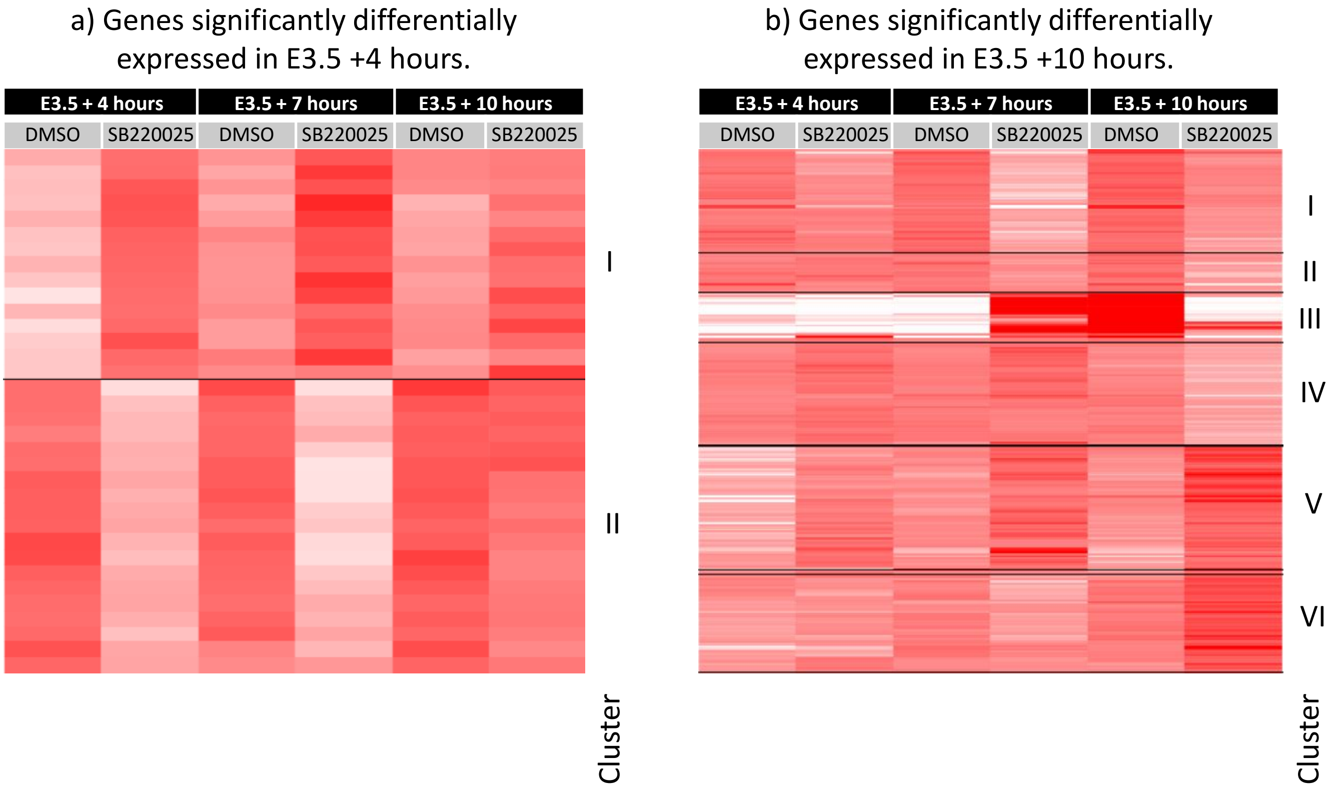

Hierarchical clustering heat-map depicting the expression of significantly changing gene mRNAs elicited by p38-MAPKi at the +4h (a) and +10h (b) time-points and the status of the transcript levels of those same genes at the two other time-points; forming two and six distinct expression clusters respectively (details in Supplementary Data 1).

Supplementary Fig. 2

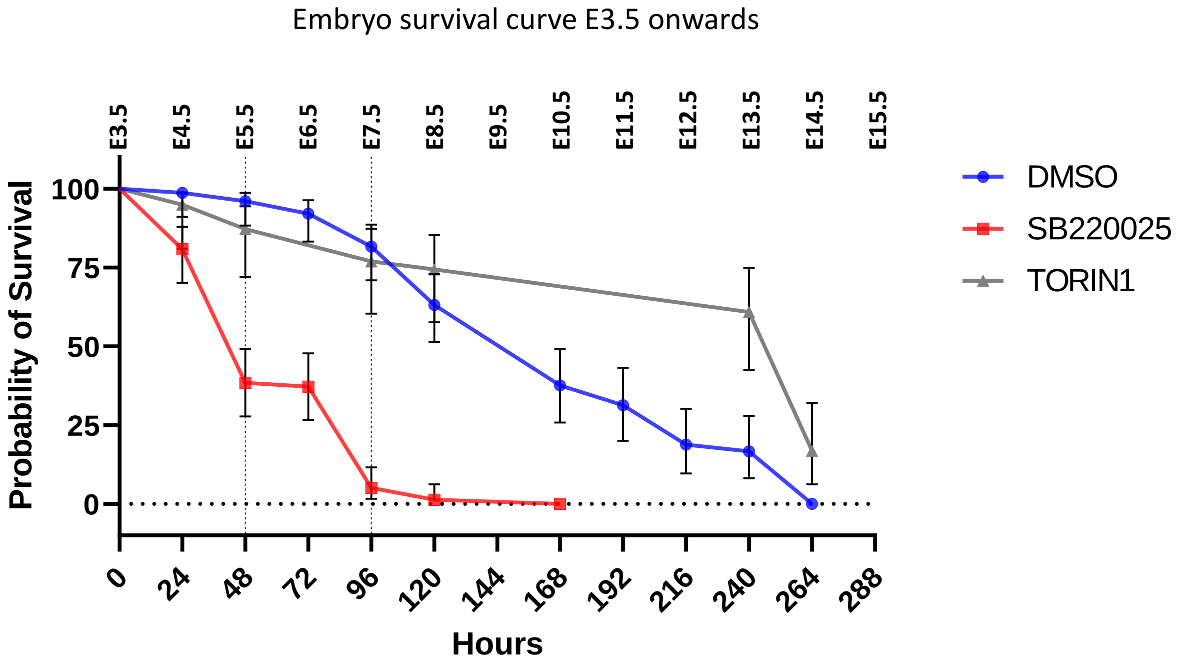

Brightfield images based analysis of survival of embryos cultured in control (DMSO; n=76), p38-MAPK inhibited (SB220025; n=78) and mTOR inhibited (TORIN1; n=39) conditions starting from E3.5.

Supplementary Fig. 3

Gene expression profile in preimplantation embryo and associated cellular lineages

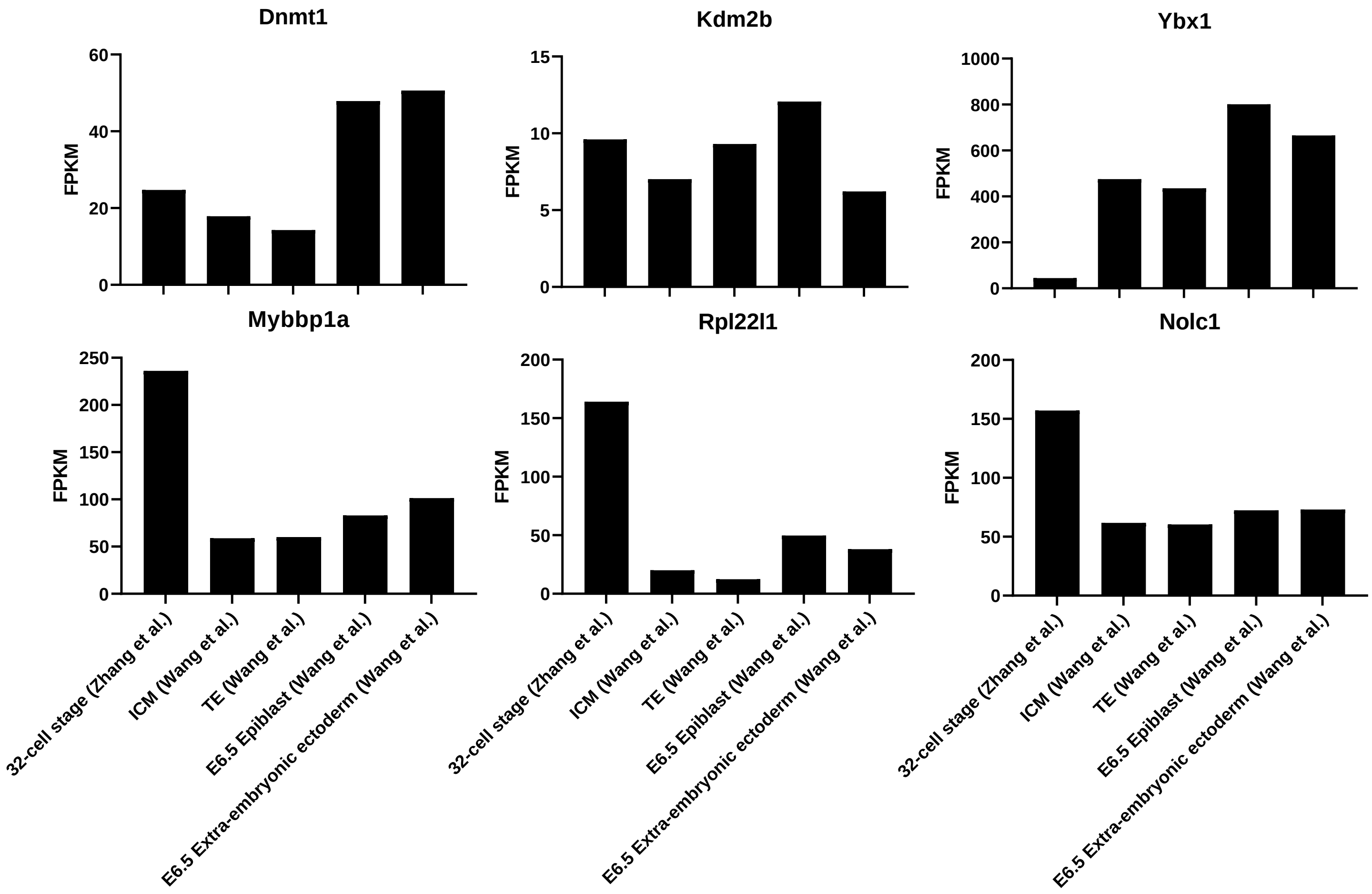

Gene expression profile of candidates identified via phosphoproteomic screen for siRNA mediated gene knockdown analysis in preimplantation embryos. Data collected from *Zhang, B., Zheng, H., Huang, B. et al. Allelic reprogramming of the histone modification H3K4me3 in early mammalian development. Nature 537, 553–557 (2016)* and *Wang, C., Liu, X., Gao, Y. et al. Reprogramming of H3K9me3-dependent heterochromatin during mammalian embryo development. Nat Cell Biol 20, 620–631 (2018).*

Supplementary Fig. 4 (supplementary to Fig. 6d-f)

Mybbp1a KD embryo cell lineage quantification (outer and inner cells and ratio of PrE to ICM).

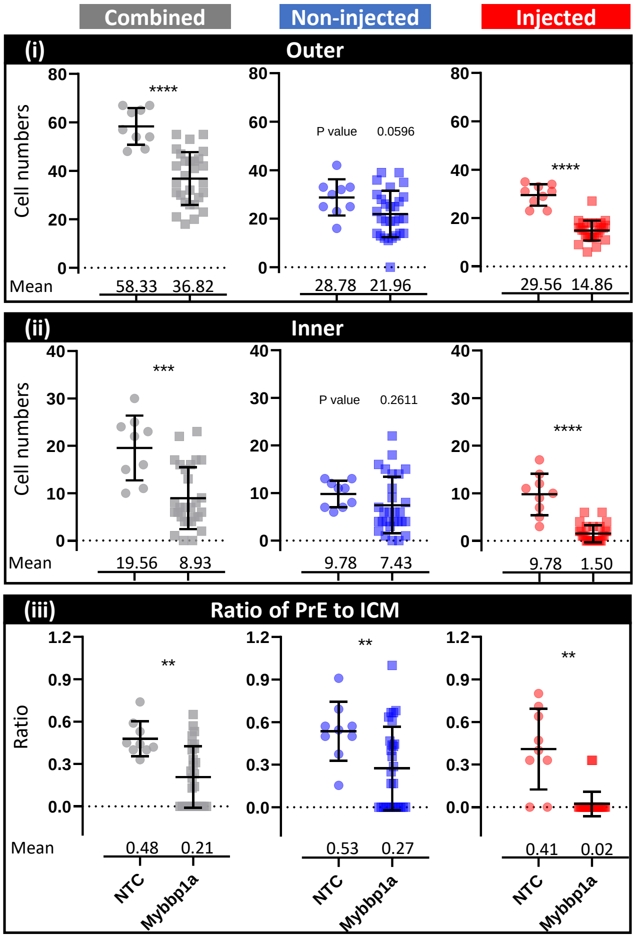

Quantification of the number of inner and outer cells and ratio of GATA4 expressing PrE to ICM upon clonal Mybbp1a knockdown. Data is supplementary to that in figure 6 (d-f).

Supplementary Fig. 5

Cellular lineage and cell numbers with addition of FGF4 (+Heparin) along with p38-MAPK inhibition.

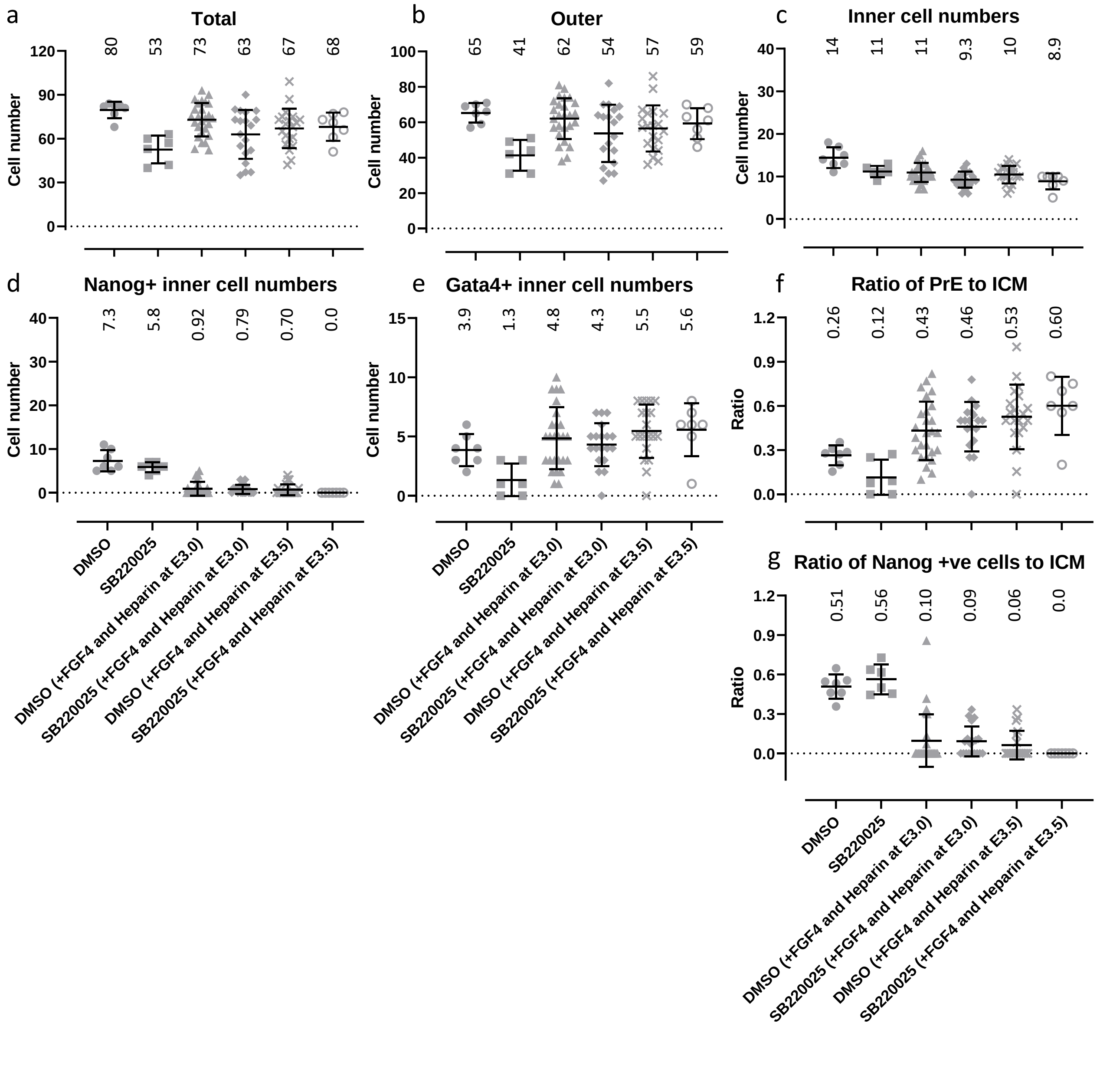

Cellular lineage and number quantification upon p38-MAPK inhibition coupled with FGF4 (+Heparin) supplementation. p38-MAPK inhibition (SB220025) and control (DMSO) treatments are beginning from E3.5, addition of FGF4 (+Heparin) was carried out at either E3.0 or E3.5. DMSO (n=7), SB220025 (n=6), DMSO + (FGF4 (+Heparin) from E3.0) (n=26), SB220025 + (FGF4 (+Heparin) from E3.0) (n=19), DMSO + (FGF4 (+Heparin) from E3.5) (n=20) and SB220025 + (FGF4 (+Heparin) from E3.5) (n=7). Tabulated cell numbers and statistical test results are in Supplementary Data1. Statistical tests used are detailed in Supplementary Table 8.

Supplementary Fig. 6

Cellular lineage and cell numbers with addition of FGF4 (+Heparin) along with MEK inhibition (PD0325901).

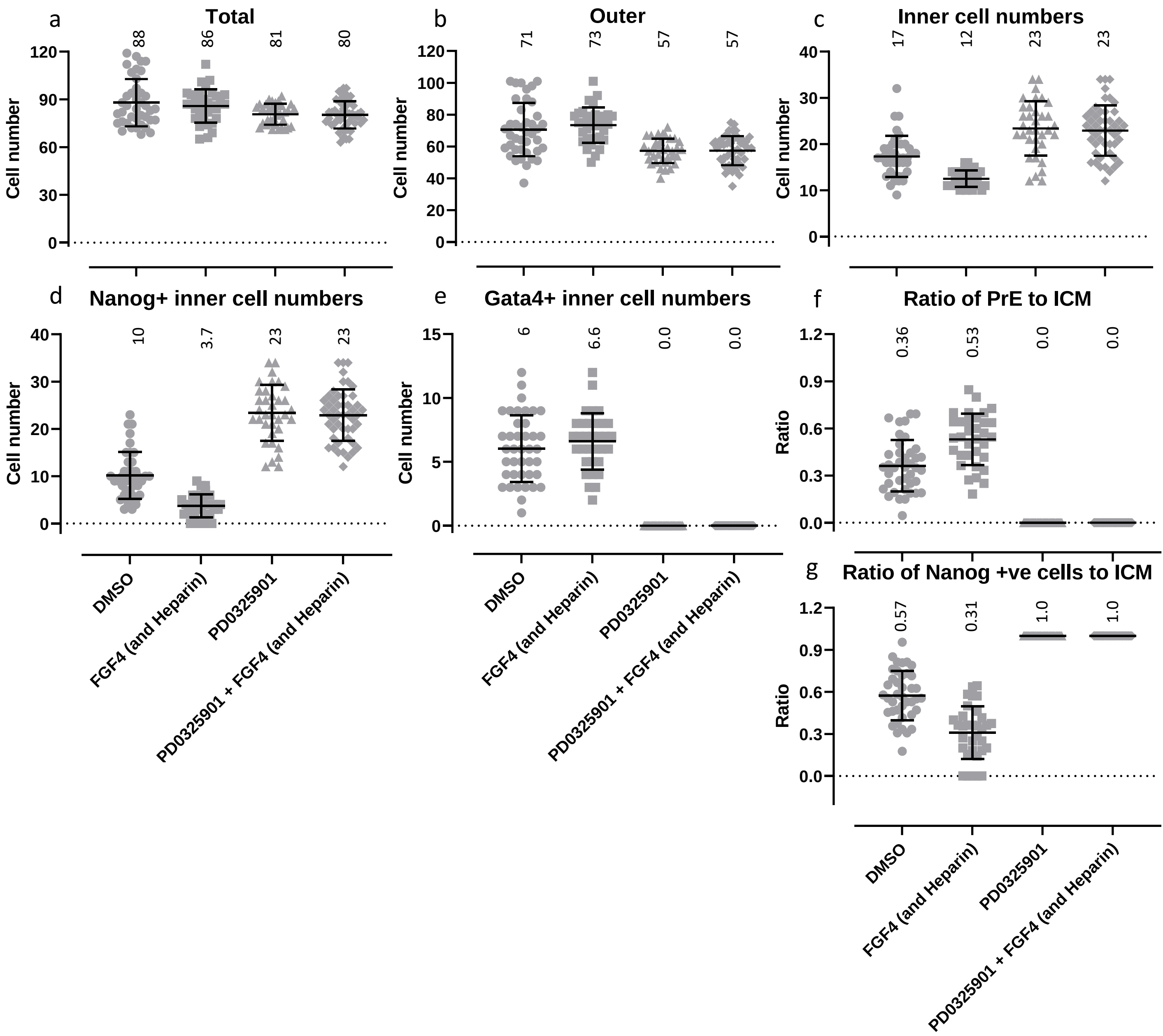

Cellular lineage and number quantification upon MEK inhibition coupled with FGF4 (+Heparin) supplementation. MEK inhibition (PD0325901) and control (DMSO) treatments are beginning from E3.5, addition of FGF4 (+Heparin) was carried out at E3.5. DMSO (n=40), FGF4 (+Heparin) (n=33), PD0325901 (n=35) and PD0325901 + FGF4 (+Heparin) (n=49). Tabulated cell numbers and statistical test results are in Supplementary Data1. Statistical tests used are detailed in Supplementary Table 8.

Supplementary Fig. 7

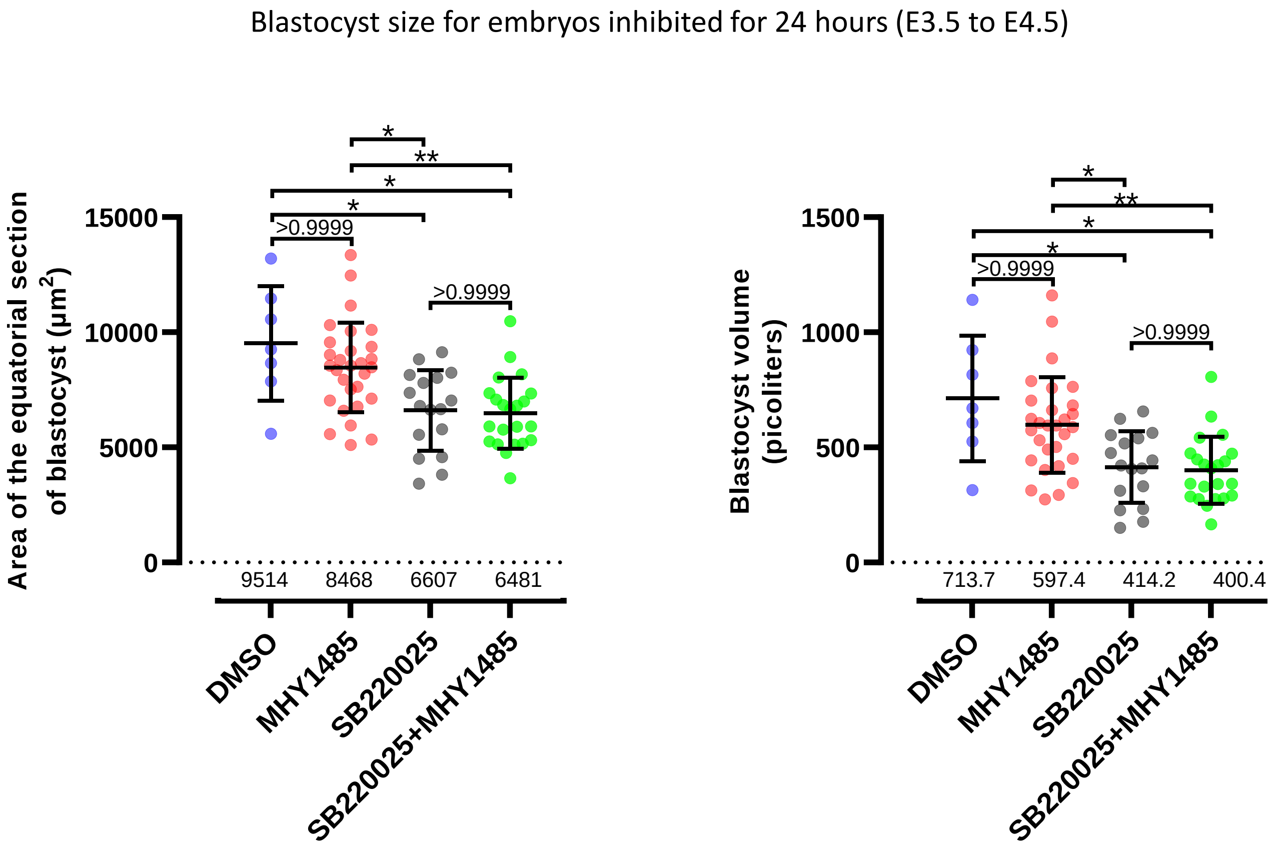

Quantification of blastocyst equatorial area (μm^2) and volume (picoliters, pL) in fixed blastocysts cultured (E3.5-E4.5) in control (DMSO; n=7), mTORa (MHY1485; n=29), p38-MAPKi (SB220025; n=17) and p38-MAPKi + mTORa (SB220025+MHY1485; n=22) conditions.

Supplementary Table 1

Atp1a1 proteome readout

Uniprot_ID	Gene Symbol	log2_FC	p-value	DMSO_1	DMSO_2	DMSO_3	p38i_1	p38i_2	p38i_3	DMSO_mean	p38i_mean
Q8VDN2	Atp1a1	0.015634914	0.891866342	25.98	25.99	25.84	25.8	25.96	26.1	25.94	25.95

Supplementary Table 2

Introns detected (as RPKM) for "translation" related genes; related to Fig. 3e and table S3e

Sl. No.	Probe	Chromosome	Start	End	Probe Strand	Feature	E3.5 +7h (DMSO)	E3.5 +7h (SB220025)
1	Rps20	4	3831334	3835665	-	null	4.335468	5.4814467
2	Rps9	7	3703993	3706897	+	null	6.2938595	8.691212
3	Rps19	7	24884371	24889806	+	null	3.595584	4.8586073
4	Rps16	7	28350652	28353155	+	null	5.842512	5.757571
5	Rps17	7	81342732	81345254	-	null	5.08963	5.088256
6	Rps3	7	99477896	99483738	-	null	10.089131	10.129799
7	Rpsa	9	120127689	120132369	+	null	5.8119106	6.4425263
8	Rps26	10	128624534	128626747	-	null	7.9579744	8.017336
9	Rps29	12	69157722	69159186	-	null	6.3753366	7.874689
10	Rps23	13	90922958	90924950	+	null	4.6760044	5.5858107
11	Rpl30	15	34440505	34443640	-	null	4.1983867	4.612251
12	Rps18	17	33951999	33956001	-	null	5.2112346	7.1981916
13	Rps14	18	60774510	60778546	+	null	7.8575892	8.69915

Supplementary Table 3

Pharmacological agents used

Sl. No.	Name	Pharmacological activity	Working concentration	Manufacturer	Cat. No.	Embryonic stage	Duration	Figures
1	SB220025	p38-MAPK inhibitor	20µM	Calbiochem	559396	E3.5 to E4.5	24 hours	1a (i), b, c, 6c, f, I, S5
2						E3.5+4 hours to E3.5+7 hours	3 hours	1a (ii)
3						E3.5 to E3.5+3 hours	3 hours	1d
4						E3.5 to E3.5+6 hours	6 hours	1d
5						E3.5 to E3.5+9 hours	9 hours	1d
6						E3.5 to E3.5+12 hours (E4.0)	12 hours	1d
7						E3.5+2 hours to E3.5+9 hours	7 hours	2
8						E3.5 to E3.5+4 hours	4 hours	3
9						E3.5 to E3.5+7 hours	7 hours	3
10						E3.5 to E3.5+10 hours	10 hours	3, 5
11	TORIN1	mTORC1/2 inhibitor	20µM	SelleckChem	S2827	E3.5 to E4.5	24 hours	6d
12	MHY1485	mTOR activator	20µM	SelleckChem	S7811	E3.5 to E4.5	24 hours	6e, h, i
13	PD0325901	MEK inhibitor	1µM	Sigma-Aldrich	PZ0162	E3.5 to E4.5	24 hours	6g, h, S5, S6
14	mFGF4	Recombinant mouse FGF4 protein (activates FGF4-MEK/ERK pathway)	1000ng/ml	R&D Systems	5846-F4	E3.0 to E4.5 and E3.5 to E4.5	36 and 24 hours respectively	S5, S6
15	Heparin sodium salt	Enhances receptor binding of FGF4	1µg/ml	Sigma-Aldrich	H3149	E3.0 to E4.5 and E3.5 to E4.5	36 and 24 hours respectively	S5, S6

Supplementary Table 4

siRNA

Sl. No.	Target (siRNA)	Working concentration	Manufacturer	Cat. No.	Assay ID
1	Negative Control	10µM	QIAGEN GeneGlobe	SI03650318	
2	Mybbp1a	10µM	Thermo Scientific Silencer Select	AM16708	156950
3	Rpl22l1	10µM	Thermo Scientific Silencer Select	4390771	s86106
4	Kdm2b	10µM	Thermo Scientific Silencer Select	4390771	s78199
5	Nolc1	10µM	Thermo Scientific Silencer Select	AM16708	287747
6	Ybx1	10µM	Thermo Scientific Silencer Select	AM16708	187614
7	Dnmt1	10µM	Thermo Scientific Silencer Select	4390771	s65072

Supplementary Table 5

Antibodies

Sl. No.	Antibody against	Type	Target	Host / isotype	Manufacturer	Catalogue number	Dilution used
**Immunofluorescence imaging**							
1	Nanog (eBioMLC-51)	Primary; monoclonal	Mouse	Rat / IgG2a	Thermo Fisher Scientific Inc. (eBioscience™)	14-5761-80	1:200 in PBST (3% BSA)
2	GATA-4 (H-112)	Primary; polyclonal	Mouse, rat and human	Rabbit / IgG	Santa Cruz Biotechnology, Inc.	sc-9053	1:200 in PBST (3% BSA)
3	GATA-6	Primary; polyclonal	Human and mouse	Goat / IgG	R&D Systems™	AF1700	1:200 in PBST (3% BSA)
4	CDX2 (CDX2-88)	Primary; monoclonal	Conserved	Mouse / IgG1, kappa	BioGenex	MU392A-5UC	1:200 in PBST (3% BSA)
5	Acetyl-Histone H4 (Lys16)	Primary; polyclonal	Mouse, rat and human	Rabbit / IgG	Merck KGaA (Sigma-Aldrich, Inc.) (Upstate®)	07-329	1:100 in PBST (3% BSA)
6	RNA polymerase II CTD repeat YSPTSPS (phospho S2)	Primary; polyclonal	Mouse, rat, human etc.	Rabbit / IgG	Abcam plc.	ab5095	1:100 in PBST (3% BSA)
7	Donkey anti-Rat IgG (H+L) Highly Cross-Adsorbed Secondary Antibody, Alexa Fluor 488	Secondary; polyclonal	Rat	Donkey / IgG	Thermo Fisher Scientific Inc.	A-21208	1:500 in PBST (3% BSA)
8	Donkey anti-Rabbit IgG (H+L) Highly Cross-Adsorbed Secondary Antibody, Alexa Fluor 555	Secondary; polyclonal	Rabbit	Donkey / IgG	Thermo Fisher Scientific Inc.	A-31572	1:500 in PBST (3% BSA)
9	Donkey Anti-Goat IgG H&L (Alexa Fluor® 647)	Secondary; polyclonal	Goat	Donkey / IgG	Abcam plc.	ab150131	1:500 in PBST (3% BSA)
10	Donkey Anti-Rabbit IgG H&L (Alexa Fluor® 647)	Secondary; polyclonal	Rabbit	Donkey / IgG	Abcam plc.	ab150075	1:500 in PBST (3% BSA)
11	Goat Anti-Rat IgG H&L (Cy3 ®) preadsorbed	Secondary; polyclonal	Rat	Goat / IgG	Abcam plc.	ab98416	1:500 in PBST (3% BSA)
12	Donkey anti-Mouse IgG (H+L) Highly Cross-Adsorbed Secondary Antibody, Alexa Fluor 488	Secondary; polyclonal	Mouse	Donkey / IgG	Thermo Fisher Scientific Inc.	A-21202	1:500 in PBST (3% BSA)

Supplementary Table 6

Primers used

		Sequence (5'->3')			
**Sl. No.**	**Primer**	**Sense**	**Antisense**	**Figures**	**Comments**
1	Tbp	GAAGAACAATCCAGACTAGCAGCA	CCTTATAGGGAACTTCACATCACAG	1d	Q-RTPCR
2	H2afz	GCGCAGCCATCCTGGAGTA	CCGATCAGCGATTTGTGGA	4b, 5e (i)	
3	Nanog	GGTTGAAGACTAGCAATGGTCTGA	TGCAATGGATGCTGGGATACTC	1d	
4	Gata4	CAAGCAGGACTCTTGGAACA	AGCAGGAATTTGAAGAGGGA	1d	
5	Mapk11	AATGTAGCGGTGAACGAGGA	CCAGATGTCCACTGTCTGGT	1d	
6	Mapk12	TATATCCATGCGGCTGGTGT	ATGTCCACTGTCTGCGTGTA	1d	
7	Mapk13	CAGCGAGGATAAGGTCCAGT	TCCAGGATCTTCAGCTCACA	1d	
8	Mapk14	ACATCGTGAAGTGCCAGAAG	CTAGGTTGCTGGGCTTTAGG	1d	
9	Mybbp1a	GTCTCTGCCGCTTCTGTCA	GGGATGATCTTAAATAGGTTTTGG	4b	
10	18S rRNA	CTCAACACGGGAAACCTCAC	CGCTCCACCAACTAAGAACG	5c, d	Q-RTPCR (SSP (from Masek et al. *Int. J. Mol. Sci.* 2020))
11	28S rRNA	CTAAATACCGGCACGAGACC	TTCACGCCCTCTTGAACTCT	5c, d	
12	45S pre-rRNA 5' ETS amplicon (1)	TCTCGCTTGTTTCTCCCGAT	GATGGGGTGGAGAGACGAG	5e, e (i)	Q-RTPCR (rRNA processing (from Corsini et al. *Cell Stem Cell* (2018))
13	45S 5' ETS & 18S overlap amplicon (2)	GCGCTTCCTTACCTGGTTG	CCGTCGGCATGTATTAGCTC	5e, e (i)	
14	18S rRNA amplicon (3)	CTGGATACCGCAGCTAGGAA	GAATTTCACCTCTAGCGGCG	5e, e (i)	
15	ITS1 amplicon (4)	GTCTCGTTTCGTTCCTGCTG	TATTTCGGGTGTGAGCGAAC	5e, e (i)	
16	5.8S rRNA amplicon (5)	GTGGATCACTCGGCTCGTG	GCAAGTGCGTTCGAAGTGTC	5e, e (i)	
17	ITS2 amplicon (6)	TCCCGAAGTTCAGACGTGTG	AGAAAGACTGGTGAGGCAGC	5e, e (i)	
18	45S ITS2 & 28S overlap amplicon (7)	CTCCTCGCTCTCTTCTTCCC	CTGTTCACTCGCCGTTACTG	5e, e (i)	
19	28S rRNA amplicon (8)	AGTAACGGCGAGTGAACAGG	GATCAGAAGGACTTGGGCCC	5e, e (i)	
