# Supplementary material for: p38-MAPK-mediated translation regulation during early blastocyst development is required for primitive endoderm differentiation in mice
Source: Commun Biol. 2021 Jun 25;4:788. doi: 10.1038/s42003-021-02290-z (PMC8233355; doi:10.1038/s42003-021-02290-z)
Supplement: Supplementary file 3 — Description of Additional Supplementary Files [file 42003_2021_2290_MOESM3_ESM.pdf]

Description of Additional Supplementary Files

File name: Supplementary Video 1

Description: Control (DMSO) E3.5 to E4.5 (first set of 16 embryos).

File name: Supplementary Video 2

Description: Control (DMSO) E3.5 to E4.5 (second set of 16 embryos).

File name: Supplementary Video 3

Description: Control (DMSO) E3.5 to E4.5 (One representative embryo magnified).

File name: Supplementary Video 4

Description: p38-MAPKi (SB220025) E3.5 to E4.5 (first set of 16 embryos).

File name: Supplementary Video 5

Description: p38-MAPKi (SB220025) E3.5 to E4.5 (second set of 16 embryos).

File name: Supplementary Video 6

Description: p38-MAPKi (SB220025) E3.5 to E4.5 (One representative embryo magnified).

File name: Supplementary Data 1

Description: Underlying source data corresponding to each figure and supplementary figure.

File name: Supplementary Data 2

Description: Contains following information:

- p38-MAPK consensus motifs: Putative p38-MAPK consensus motifs and/or reported p38-MAPK interaction for phosphopeptides identified.
- Additional candidate KDs: Additional phosphoproteome candidate knockdown.
- Statistical tests: Statistical tests.
