# Supplementary material for: p38-MAPK-mediated translation regulation during early blastocyst development is required for primitive endoderm differentiation in mice
Source: Commun Biol. 2021 Jun 25;4:788. doi: 10.1038/s42003-021-02290-z (PMC8233355; doi:10.1038/s42003-021-02290-z)
Supplement: Supplementary file 12 — Reporting Summary [file 42003_2021_2290_MOESM12_ESM.pdf]

nature research

Corresponding author(s): Pablo Bora & Alexander W. Bruce

Last updated by author(s): May 7, 2021

Reporting Summary

Nature Research wishes to improve the reproducibility of the work that we publish. This form provides structure for consistency and transparency in reporting. For further information on Nature Research policies, see our Editorial Policies and the Editorial Policy Checklist.

Statistics

For all statistical analyses, confirm that the following items are present in the figure legend, table legend, main text, or Methods section.

n/a	Confirmed	
☐	☒	The exact sample size (*n*) for each experimental group/condition, given as a discrete number and unit of measurement
☐	☒	A statement on whether measurements were taken from distinct samples or whether the same sample was measured repeatedly
☐	☒	The statistical test(s) used AND whether they are one- or two-sided<br>*Only common tests should be described solely by name; describe more complex techniques in the Methods section.*
☒	☐	A description of all covariates tested
☐	☒	A description of any assumptions or corrections, such as tests of normality and adjustment for multiple comparisons
☐	☒	A full description of the statistical parameters including central tendency (e.g. means) or other basic estimates (e.g. regression coefficient) AND variation (e.g. standard deviation) or associated estimates of uncertainty (e.g. confidence intervals)
☐	☒	For null hypothesis testing, the test statistic (e.g. *F*, *t*, *r*) with confidence intervals, effect sizes, degrees of freedom and *P* value noted<br>*Give P values as exact values whenever suitable.*
☒	☐	For Bayesian analysis, information on the choice of priors and Markov chain Monte Carlo settings
☒	☐	For hierarchical and complex designs, identification of the appropriate level for tests and full reporting of outcomes
☒	☐	Estimates of effect sizes (e.g. Cohen's *d*, Pearson's *r*), indicating how they were calculated

Our web collection on statistics for biologists contains articles on many of the points above.

Software and code

Policy information about availability of computer code

Data collection	Fluoview (Olympus) 10, BioRad CFX Manager, PrimoVision (Vitrolife).
Data analysis	Microsoft Office Excel, GraphPad Prism 8, FIJI Image J, Bitplane Imaris, Fluoview (Olympus) 10, BioRad CFX Manager, Trim Galore! v0.4.1, Hisat2 v2.0.5, DESeq2 within Seqmonk v1.45.4, GOrilla.

For manuscripts utilizing custom algorithms or software that are central to the research but not yet described in published literature, software must be made available to editors and reviewers. We strongly encourage code deposition in a community repository (e.g. GitHub). See the Nature Research guidelines for submitting code & software for further information.

Data

Policy information about availability of data

All manuscripts must include a data availability statement. This statement should provide the following information, where applicable:

- Accession codes, unique identifiers, or web links for publicly available datasets
- A list of figures that have associated raw data
- A description of any restrictions on data availability

RNA-seq datasets are available in GEO database with accession number GSE162233. The mass spectrometry proteomics data have been deposited to the ProteomeXchange Consortium via the PRIDE partner repository with the dataset identifier PXD025711.

Field-specific reporting

Please select the one below that is the best fit for your research. If you are not sure, read the appropriate sections before making your selection.

☒ Life sciences ☐ Behavioural & social sciences ☐ Ecological, evolutionary & environmental sciences

For a reference copy of the document with all sections, see nature.com/documents/nr-reporting-summary-flat.pdf

Life sciences study design

All studies must disclose on these points even when the disclosure is negative.

Sample size	Samples sizes are in accordance with general practice in the field and previous publications from the lab.
Data exclusions	No data was excluded.
Replication	Biological and technical replicates were performed.
Randomization	Embryos used for microinjections and inhibitor treatment were randomly picked from a pool of naturally fertilized embryos. Further experiments were carried out from the said inhibitor or control treated embryos with no exclusions.
Blinding	The researchers were not blinded during the experiment or analysis.

Reporting for specific materials, systems and methods

We require information from authors about some types of materials, experimental systems and methods used in many studies. Here, indicate whether each material, system or method listed is relevant to your study. If you are not sure if a list item applies to your research, read the appropriate section before selecting a response.

Materials & experimental systems

n/a	Involved in the study	
☐	☒	Antibodies
☒	☐	Eukaryotic cell lines
☒	☐	Palaeontology and archaeology
☐	☒	Animals and other organisms
☒	☐	Human research participants
☒	☐	Clinical data
☒	☐	Dual use research of concern

Methods

n/a	Involved in the study	
☒	☐	ChIP-seq
☒	☐	Flow cytometry
☒	☐	MRI-based neuroimaging

Antibodies

Antibodies used	Described in details within supplementary tables.
Validation	All the antibodies were commercially available and the validation of each antibody was confirmed by the manufacturers' websites.

Animals and other organisms

Policy information about studies involving animals; ARRIVE guidelines recommended for reporting animal research

Laboratory animals	F1 hybrid (C57Bl6 x CBA/W) females and F1 males were used for mating and 2-cell stage embryos isolated from the F1 hybrid females were used for experiments.
Wild animals	Study did not involve wild animals.
Field-collected samples	Study did not involve samples collected from field.
Ethics oversight	All mouse related experimental procedures (i.e. collecting preimplantation stage embryos for further study) complied with 'ARRIVE' guidelines and were carried out in accordance with EU directive 657 2010/63/EU (for animal experiments).

Note that full information on the approval of the study protocol must also be provided in the manuscript.
